# In Vivo Evaluation of Histopathological Alterations and Trace Metals Estimation of the Small Intestine in Bisphenol A-Intoxicated Rats

**DOI:** 10.1155/2019/9292316

**Published:** 2019-12-09

**Authors:** Saira Ambreen, Tasleem Akhtar, Naila Hameed, Isbah Ashfaq, Nadeem Sheikh

**Affiliations:** ^1^Cell and Molecular Biology Lab, Department of Zoology, University of the Punjab, Lahore, Pakistan; ^2^Department of Pharmacology, University of Health Sciences, Lahore, Pakistan

## Abstract

BPA, a ubiquitously used plasticizer, has become one of the contaminants of emerging concern and causes many serious health implications in humans due to multiple exposure pathways. The current study was aimed at investigating the deformities of structures that arise by exposure of the small intestine to BPA through trace elements estimation of tissues as well as the study of serum profile. Two major groups of Wistar rats were established: one control group and the other experimental group, which was further divided into four groups based on dose (10 mg/kg/bodyweight and 25 mg/kg/bodyweight, respectively) and duration of exposure (6 and 12 weeks, respectively). Histological study of the small intestine showed the distorted structures in the experimental groups. The special staining performed illustrated the accumulation of calcium deposits in the small intestinal tissue in treated groups. Trace metals estimation showed a significant increase in the metallic content of sodium and iron and a decrease in the calcium content in the experimental groups (*p*=0.05). Serum profiling illustrated an increase in total iron-binding capacity and glucose levels and a decrease in the serum total iron level (*p*=0.05). An increased expression of a proinflammatory cytokine (IFN-*α*) was observed in the liver. From all these findings, it was inferred that BPA caused many structural alterations in the small intestinal tissue, which further affected its functioning. The calcium deposits seen through special staining affected the motility of the small intestine and caused its dysfunction. It was also induced from serum profiling that BPA affected the homeostasis of iron and glucose and caused its imbalance. Also, as BPA got absorbed from the small intestine and reached the liver via the blood stream, it caused hepatoxicity in the liver and led to increased inflammatory response by IFN-*α* against the toxicant.

## 1. Introduction

Bisphenol A (2,2-bis (4-hydroxyphenyl) propane), one of the major contaminants of emerging concern (CEC), is a highly consumed and produced chemical worldwide [1, 2]. BPA is a plastic monomer that is used as an intermediate in binding, plasticizing, and hardening polycarbonate plastics, polyester-styrene, and epoxy resins. High thermal stability and good mechanical properties make BPA a widely used chemical in daily life products of different kinds including dish and laundry detergents, digital media, medical devices, care products, reusable bottles (e.g., baby bottles), water dispensers, adhesives, tub and tiles [2–4], sports safety equipment, food storage containers, and children toys. BPA is mainly used as an internal lining of the food and beverage containers to prevent the contact of food and drinks with metallic surface [2, 3, 5].

Bisphenol A (BPA) is a man-made compound with high prevalence in our environment and is suspected to be an endocrine disruptor [6]. Human population is being exposed to BPA inexorably and continuously via ingestion of food and drinking water, inhalation of ambient and indoor air, dust, and through direct contact with products containing the compound. Out of all different exposure pathways to humans, diet acts as a primary source of exposure [2, 7]. Human studies regarding BPA toxicity have shown adverse and irreversible effects on human health. Mainly effects produced by BPA include reproductive defects in both male and females. In females, increased level of BPA leads to genetic defects, e.g., Down's syndrome and persistent miscarriages and increased number of premature deliveries; however, detrimental effects have not yet been proved in ovum cells in humans [6, 8, 9]. In males, BPA effects include anomalies of genitalia and spermatogenesis [10]. Prenatal exposure to BPA may bring hyperactivity and increased aggression at 2 years of age, primarily in females. Other purported effects reported by scientists include cardiovascular diseases, diabetes, altered behavior, hyperinsulinemia, mal-immune response increased adiposity (obesity), affected brain/sexual differentiation, neoplastic, and preneoplastic lesions (mammary and prostate) [11].

The current study was designed to investigate the deformities of structures that arise by exposure of the small intestine to BPA by using histopathological techniques along with trace elements estimation of tissues as well as the study of serum profile and hepatic gene expression involved in inflammatory response against BPA.

## 2. Materials and Methods

### 2.1. Animals Preparation

Male Wistar rat colonies were reared in the animal house of Department of Zoology (University of the Punjab) under standard laboratory conditions and were provided proper access to food and water ad libitum.

### 2.2. Experimental Design

The rat colonies were divided into five groups with seven animals in each group. All the rats were assigned to the cages using the balloting method. One was negative control while the rest of four experimental groups were given low dose of BPA (10 mg/kg/bodyweight) and high dose of BPA (25 mg/kg/bodyweight) for 6 and 12 weeks, respectively. These are low-dose six-week (L-6), low-dose twelve-week (L-12), high-dose six-week (H-6), and high-dose twelve-week (H-12) experimental groups. BPA was purchased from Sigma and given to rats orally dissolved in corn oil through oral gavage.

### 2.3. Serum TIBC, TI, and RBG Estimation

For in vitro quantification of serum total iron binding capacity (TIBC), total iron (TI), and random blood glucose (RBG), prepared commercial kits TIBC Cat No. TI1010 (by Randox) kit, Fer-color. No. 1492001 (by Wiener Lab 2000 Rosario-Argentina), and Chemhouse (Cat No. 050-0300) were used, respectively.

### 2.4. Histochemical Trace Metals Estimation

0.25 g of the intestinal tissue was taken following the addition of 2.5 ml of HNO_3_ and boiled at 90–95°C for 10–20 minutes. Then, H_2_O_2_ (0.75 ml) and deionized water (0.5 ml) were added and boiled again to stop effervescence. After that, H_2_O_2_ was added until the solution became transparent. Finally, deionized water was used to make up the final volume of 25 ml. For metal detection, solution was run directly in a Perkin Elmer atomic spectrophotometer (Model 800). Hollow cathode lamp was equipped with the spectrophotometer and selected per analytes.

### 2.5. Hematoxylin and Eosin (H&E) Staining Technique for Morphological Changes

Small intestinal tissues collected from sacrificed animals were fixed in 10% neutral buffered formalin and then processed through ascending grades of ethanol and xylene before imbedding in paraffin block. The paraffin-embedded intestine tissues were cut into 3 *μ*m sections. Deparaffinized sections were hydrated with ethanol grades. These hydrated sections were incubated in Harris hematoxylin for 2.5 minutes followed by washing in running tap water. Sections were counterstained with eosin. Sections were dehydrated with ethanol and washed with xylene. The histopathological variations of the small intestine were observed under a light microscope.

### 2.6. Von Kossa's Staining Technique for Calcium Deposition

Von Kossa's staining technique was used to detect cytoplasmic deposition of calcium in intestinal (duodenal part) smooth muscle cells and extracellular spaces of the muscularis externa. The 3–5 *μ*m thick tissue sections were processed with descending grades of ethanol for hydration. These sections were placed under a 100 watt lamp for 45–60 minutes subsequently dipping in 5% silver nitrate solution. Sections were rinsed in distilled water and treated with 5% sodium thiosulphate solution for 5 minutes. Afterwards, the sections were counterstained with 1% neutral red solution. Sections were dehydrated by washing with ascending grades of ethanol and cleared with xylene. Images were taken using a light microscope.

### 2.7. Estimation of Hepatic IFN-*α* Expression

Total RNA was extracted by using TRIzol and chloroform and quantified by using optical density measurement (260/280 nm). RNA integrity was checked by using agarose gel electrophoresis. The mRNA reverse transcription was performed using the Oligo (dT) 18 primer and RevertAid. cDNA made was used as a template and PCR-amplified gene-specific primers. The primer pairs used were as follows: IFN-*α*: (forward) 5′-AGG TAG GGG TGC AGG AAT CT-3′ and (reverse) 3′-GCA CAG GGG CTG TGT TTA TT-5′; *β*-actin: (forward) 5′-TGT CAC CAA CTG GGA CGA TA-3′ and (reverse) 3′-AAC ACA GCC TGG ATG GCT AC-5′. PCR was performed on Thermo Cycler of Labinet (Model # MultiGene OptiMax) and forty cycles were repeated, and the amplified products were resolved on 2% agarose gel loaded with ethidium bromide and visualized on a UV transilluminator. The gel was later analyzed using image J software and was normalized to that of *β*-actin housekeeping gene.

### 2.8. Statistical Analysis

Results were analyzed using GraphPad Prism 5 by applying one-way ANOVA following Tukey's post hoc test for multiple comparison. *p* value of 0.05 was considered significant. Data were represented in the form of mean ± SEM.

## 3. Results

### 3.1. BPA-Induced Changes in Serum TIBC, TI, and RBG

**Figure 1:**
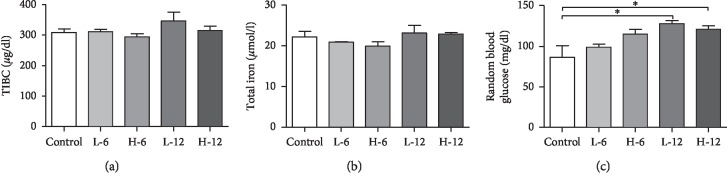
Serum analysis for assessment of damage. (a) Total iron binding capacity, (b) total iron content, and (c) random blood glucose level showing damage due to BPA doses. Statistical analysis was performed using one-way ANOVA with Tukey's post hoc test (*p* ≤ 0.05).

The serum biochemical analyses such as TIBC and TI content showed insignificance in the experimental groups compared to the control group (Figures 1(a) and 1(b)), respectively. However, when compared to RBG in serum, there was significant alteration in low and high doses of BPA for the twelve-week experimental group in contrast to the control group at *p* ≤ 0.05, shown in Figure 1(c).

### 3.2. Significant Alteration in Serum Essential Trace Metals

Serum metallic concentration is the most commonly used diagnostic factor in determining gastrointestinal toxicity.

#### 3.2.1. Calcium

**Figure 2:**
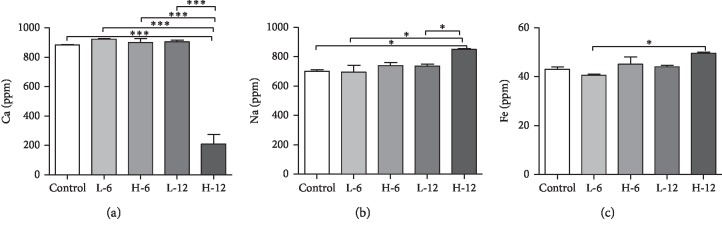
Effect of oral administration of BPA on the change in the levels of metallic content: (a) Ca, (b) Na, and (c) Fe. Each value is represented as mean ± SEM for each experimental group in contrast to the control group. ^*∗*^*p* ≤ 0.05 , ^*∗∗*^*p* < 0.01, and ^*∗∗∗*^*p* < 0.001.

After exposure to BPA, there was a significant decline of serum calcium observed in the twelve-week high-dose BPA-treated group in contrast to low-dose BPA and high-dose BPA for the six-week experimental group, low-dose BPA for the twelve-week experimental group, and the control group, as shown in Figure 2(a).

#### 3.2.2. Sodium

The level of sodium increased significantly in high-dose BPA for the twelve-week experimental group in contrast to the low-dose BPA for the six-week experimental group and control group (Figure 2(b)).

#### 3.2.3. Iron

The level of iron in serum significantly increased in the high-dose twelve-week experimental group compared to the low-dose six-week experimental group, as shown in Figure 2(c).

### 3.3. BPA Caused Intestinal Tissue Impairment in Rats

**Figure 3:**
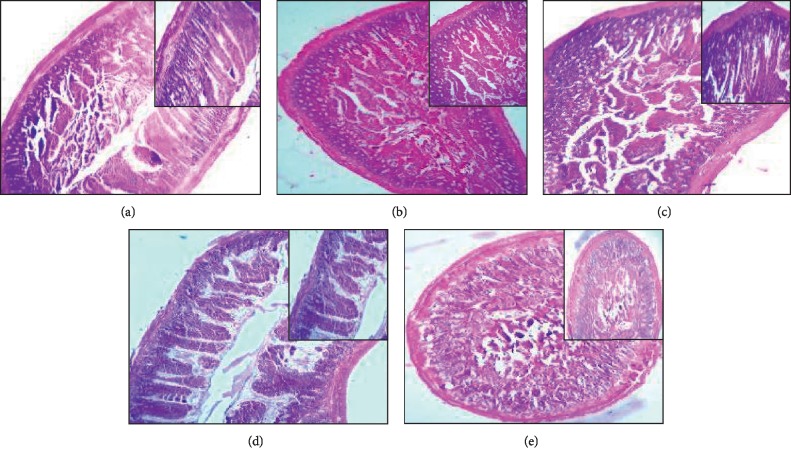
Intestinal tissue stained with H&E showed significant impairment in the experimental groups in contrast to the control group at 4x and 10x. (a) Control; (b) L-6; (c) H-6; (d) L-12; (e) H-12.

Histological analysis illustrated the impairment of the small intestinal tissue of rat groups treated with BPA compared with control (Figure 3).  Figure 3(a) of the control group showed normal intestinal epithelium lined by columnar cells and thrown into villi in contrast to the experimental groups. The low-dose six-week BPA-treated group showed minor injury in the small intestine. However, compared to the high-dose six-week group, there was significant disruption in the intestine; for example, hyperplasia of the cells was observed in the lamina propria along with shrinkage and rupturing of the villi. Lesions in the villus epithelium were quite discernible. Necrosis of mucosa and submucosa was also found. The low-dose twelve-week BPA-treated group showed infiltration of neutrophils in the villi epithelium and lamina propria, and villi with flattened tips were observed. Shrunk lacteal, necrotic epithelium, and ruptured goblet cells were also detected. There was a significant increase of intestinal disruption in the high-dose twelve-week BPA-treated group in contrast to the low-dose 12-week BPA-treated group.

### 3.4. BPA Increased Calcification in the Small Intestine

**Figure 4:**
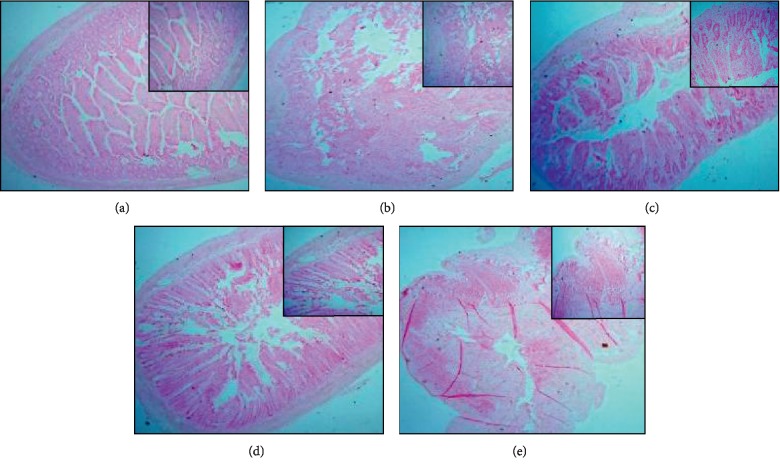
Von Kossa's staining for histological visualization of calcium salt deposits at 4x and 10x showed significant dark deposition of calcium salt in the experimental groups in contrast to the control group. (a) Control; (b) L-6; (c) H-6; (d) L-12; (e) H-12.

Von Kossa's staining for detection of calcium deposition in the small intestine showed deposits of calcium in the experimental groups in contrast to the control group. In Figure 4, the high-dose BPA groups showed more calcium salt in the submucosal and muscularis layer of the small intestine in comparison with the low-dose BPA-treated groups.

### 3.5. Expression Regulation of Hepatic IFN-*α*

**Figure 5:**
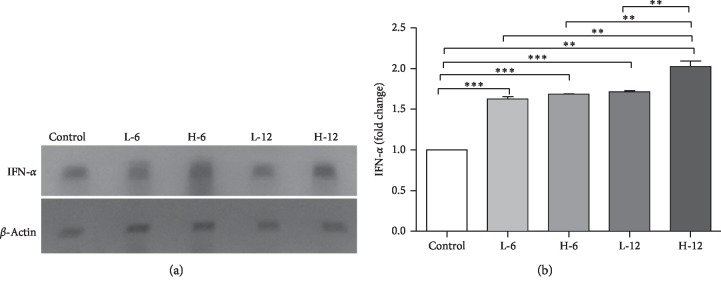
Effect of BPA on expression regulation of IFN-*α*. (a) The illustration of the conventional PCR product of IFN-*α*. (b) The fold changes in the expression of the hepatic IFN-*α* mRNA following statistical analysis. All groups showed increased trend with the highest level in the high-dose twelve-week BPA exposure group (^*∗*^significance at *p* ≤ 0.05, ^*∗∗*^significance at *p* ≤ 0.01, and ^*∗∗∗*^significance at *p* ≤ 0.001).

The expression regulation of hepatic IFN-*α* showed significant upregulation in the experimental groups compared to the control group. The increased expression regulation of IFN-*α* in all the experimental groups was shown as fold changes, i.e., 0.63 fold ± 0.030 in the low-dose six-week BPA-treated group, 0.68 fold ± 0.003 in the high-dose six-week BPA-treated group, and 0.71 fold ± 0.014 in the low-dose twelve-week BPA-treated group, with the highest level in the high-dose twelve-week group (1.02 fold ± 0.066) when analyzed by one-way ANOVA followed by Tukey's post hoc test at significance *p* ≤ 0.05, *p* ≤ 0.01, and *p* ≤ 0.001, as shown in Figures 5(a) and 5(b).

## 4. Discussion

BPA is one of the highly produced chemicals with its worldwide distribution in air, sediments, soils, surface waters, effluent discharge, sewage sludge, biosolids, wildlife, and humans [12–14]. It has been reported that BPA disturbs the normal endocrine functions and causes adverse effects on human health such as cancer, immune system disorders, and malformations of the reproductive system [15]. The purpose of the current study was to evaluate the histochemical changes caused by BPA on the small intestine and the effect of BPA on the levels of sodium, calcium, and iron (Na, Ca, and Fe) in the intestine and also to investigate the serum levels of total iron, TIBC, and glucose. PCR was performed to determine the changes in the hepatic gene expression of IFN-*α*.

H&E staining performed to evaluate the histological changes showed the sections with the epithelial necrosis, mucosal and submucosal lesions, goblet cells loss, hyperplasia of lamina propria, and neutrophilic infiltration in the experimental groups in comparison to the control group. These results were supported by the study of other toxicants effect on the small intestine that showed degeneration of intestinal epithelium, increased hypertrophy of cells, epithelial desquamation from lamina propria, atrophy of the villi, basophilic infiltration, and degeneration of the intestinal glands [16].

In the current study, Von Kossa's staining was performed to illustrate the deposition of calcium in the small intestinal tissue in treated groups. Calcium deposition in the small intestinal submucosal and muscularis smooth muscle layer leads to the dysfunction of intestinal motility which is caused as a consequence of depression of myoneural transmission at synapses or inhibition of cross bridges formation in smooth muscles due to the nonavailability of calcium because of its calcification. These results correlate with the studies where animals fed with BPA had high levels of calcium deposits in intestinal mucosa and submucosa [17].

The small intestine plays a pivotal role in the iron homeostasis of the body and the exposure of humans to iron is mainly through diet, so BPA can affect the iron absorption in the small intestine [18]. Our results also reported that an increase in the BPA exposure causes more iron absorption into the intestinal tissue than into the blood circulatory system. Serum profiling also supported the result that high-dose exposure of BPA leads to low iron levels and leads to anemia. Iron deficiency being a confounding factor in inducing oxidative stress leads to the redox reaction of the hemoglobin with RBCs under hypoxic condition, produces changes in its membrane structure, and triggers their removal from circulation. Hence, oxidative stress leads to anemic condition [13, 14, 19, 20].

Luminal sodium plays a major role in intestinal absorption of glucose. Trace metals study also revealed that BPA exposure for a longer duration (12 weeks) stimulated the accumulation of more sodium in the small intestine which in turn caused the more absorption of glucose in the blood that may lead to hyperglycemia and type-2 diabetic condition in a longer run [21, 22]. These results were in accordance with the serology results, which indicated the increased glucose profile in the serum of exposed adult male rats after a long-term exposure of 12 weeks.

The effect of BPA on the calcium content in the small intestine demonstrated that a decrease in the calcium level on high-dose exposure to BPA forms lesions in the duodenal smooth muscle layer by forming calcium deposits, which lead to less availability of calcium in the efferent terminals of local myenteric Auerbach's plexuses of the smooth muscle layer. This result is also supported by the staining of the small intestine for the calcium deposition, which indicates the deposition of calcium is high for high levels of BPA, as suggested by Sarkar et al. [23].

Our results showed a decreased trend of total iron content for L-6 and H-6 groups and an increased trend for the L-12 group, while no significant change was observed for the H-12 group as compared to the control. Free iron in the serum (nontransferrin-bound iron) produces ROS in Fenton reaction and that ROS produces oxidative stress and leads to further diabetic complexities [24]. The oxidative stress is reported to modulate several physiological functions of cells such as signal transduction. It is also believed to be cytotoxic, leading to cells and organs damage. Besides that, oxidative stress is believed to cause hemolytic anemia by causing ineffective erythropoiesis [25]. These results were also supported by the literature, corresponding to which EDCs cause decelerating erythropoiesis by suppressing EPO induction, as a result anemia develops [26].

Our results showed an increased trend for TIBC in all the experimental groups with peak value attained in the low-dose twelve-week group, and a probable explanation obtained from the literature is that enhanced TIBC level in serum is caused by increased iron retention in the tissue as illustrated by the work done on monosodium glutamate [27].

When a toxicant interacts with the small intestine, it can not only damage the organ but also gets metabolized through the actions of biotransformation enzymes. A fraction of the metabolites gets transported across the gut epithelium, absorbed into the blood stream, and reach the liver. In the liver, biotransformation enzymes (cytochrome P450) further metabolize chemicals and other xenobiotics such as BPA, which results in hepatotoxicity of the liver [28, 29]. Our results showed the evidence of proinflammatory response by demonstrating increased expression of cytokine IFN-*α* in all the experimental groups as compared to control. These results demonstrated that the proinflammatory response towards BPA was significant by IFN-*α*, and hence, IFN-*α* mediated the immune response towards BPA toxicity. BPA affects the SOCS-3 secretion and insulin signal transduction by decreasing the expression of SOCS-3 mRNA and its proteins. A decreased level of SOCS proteins promotes increased expression of type-1 IFNs that result in different types of complexities [30].

## 5. Conclusion

All the present findings lead us to the conclusion that BPA leads to impairment of the small intestinal architecture. Inflammatory actions of BPA on the small intestine were confirmed by the intestinal lesions, epithelial necrosis, neutrophilic infiltrations, and disruption of villi structure. Likewise, BPA-induced alterations in trace elements of the small intestine revealed its inflammatory character. Gene expression of IFN-*α* showed marked changes affected by BPA. Changes in the serum level of total iron, TIBC, and glucose confirmed the major complexities caused by BPA intake.

## Data Availability

The data used to support the findings of this study are available from the corresponding author upon request.
